# Enumerating Antibiotic Susceptibility Patterns of *Pseudomonas aeruginosa* Isolated from Different Sources in Dhaka City

**DOI:** 10.2174/1874285801812010172

**Published:** 2018-05-31

**Authors:** Mahmudullah Bhuiya, Mohammad K. I. Sarkar, Mehadi H. Sohag, Hafij Ali, Chapol K. Roy, Lutfa Akther, Abu F. Sarker

**Affiliations:** 1 Stamford University, Dhaka, Bangladesh; 2 Independent University, Bangladesh (IUB), Bashundhara R/A, Dhaka-1212, Bangladesh; 3 Jagannath University, Dhaka, Bangladesh; 4 Bangladesh Council of Scientific and Industrial Research (BCSIR), Dhaka, Bangladesh; 5 Dhaka Medical College Hospital, Bangladesh; 6 Rajshahi University, Dhaka, Bangladesh

**Keywords:** *Pseudomonas aeruginosa*, Different sources, Antibiotic susceptibility, Resistant, Environmental isolates, Gram-negative bacteria

## Abstract

**Background:**

*Pseudomonas aeruginosa* is a ubiquitous free-living bacterium and is responsible for severe nosocomial infections, life-threatening infections in immune compromised persons. The bacterium, along with its natural resistance, can acquire resistance to many antibiotics by a variety of methods.

**Method:**

Therefore, to compare the antibiotic sensitivity pattern of *Pseudomonas aeruginosa*, a total of seventeen isolates of *P. aeruginosa* were isolated from different sources; for example environmental sources, frozen food sources, clinical sources and medical waste materials. Isolates were confirmed to be *P. aeruginosa* by cultural and biochemical properties.

**Result:**

The isolates were tested against seventeen commercially available antibiotics to observe the antibiotic susceptibility patterns. Imipenem and meropenem were the most potent antibiotics (100% sensitivity) followed by amikacin and piperacillin with maximum sensitivity. Among others, gentamicin, ciprofloxacin, levofloxacin and aztreonam were found to be fairly active. A good number of isolates were intermediately resistant to ceftriaxone. The rates of resistance to aztreonam, cefotaxime and ceftazidime were 11.76%, 82.35% and 5.88% respectively. Complete resistance was observed against penicillin, ampicillin, cefixime and cefpodoxime.

**Conclusion:**

It can be concluded that the clinical isolates including isolate from medical waste, were multi-drug resistant than environmental and food isolates indicating the risk of transmission of resistance to the environmental isolates of *P. aeruginosa*.

## 1 INTRODUCTION

The ability to cause disease in particular susceptible individuals as well as environmental versatility makes *Pseudomonas aeruginosa* a unique one among gram-negative bacteria pool that is frequently availed in small numbers in intestinal flora and on the integument portions of human body [1].

Hence, with significant capability to adapt to nutritionally challenged condition and energy uptake limitations, *P. aeruginosa* has shown notable competency to spread out from soil and water sources to living organisms [2, 3]. Nonetheless, *P. aeruginosa* intensively interferes in human immune mechanisms through producing a number of toxic macromolecules, where extensive tissue damage is also an outcome. Resistance to antibiotics is also a notion for this bacterium, which opens a window for scientific community to study the susceptible nature of *P. aeruginosa* in response to antibiotics along with metabolic performance in unlike conditions [4]. Cutting-edge research on genomics science has revealed the genome sequence of *P. aeruginosa* that has been analyzed to identify genes involved in locomotion, antibiotic efflux, transport and utilization of nutrients and responding in different environmental alteration [5-7].

From the last century, *P. aeruginosa* has been treated as one of the topmost hospital pathogens and availed as opportunistic bacteria in recurrent infected patients admitted to the hospital along with frequent places of hospital environment [8, 9]. It is affirmed that *P. aeruginosa* is ranked as the fifth common clinical microbes that are responsible for one-tenth portion of attained infections in hospitals. In case of Bangladesh, this scenario is more dreadful and it causes a wide range of infections [10]. However, the regretting fact is that, this bacterium has become increasingly resistant to various antimicrobial agents. Additionally, waste materials released from the healthcare centers would also be considered as a potential source of drug-resistant *P. aeruginosa* that can be spread to the environment [11].

The present study was designed to enumerate the comparison of antibiotic susceptibility pattern of *P. aeruginosa* isolated from different environmental samples, medical waste and food sources against some commonly used antibiotics.

## 2 METHODS AND MATERIALS

### 2.1 Site, Source and Collection of Sample

As the study was based on Dhaka city, the capital of Bangladesh, selected samples were taken from different parts of Dhaka City Corporation and notably from Dhanmondi, Siddeshwari, Kamalapur, Khilgaon, Bashaboo, Kamrangirchar, Babubazar, Mughdapara, Manda, Shahbagh, Ramna and so on. To isolate *Pseudomonas aeruginosa*, a total of seventeen samples were collected from different sources *e.g.*, soil, water, food and healthcare center. Among all, two samples were isolated from soil sources, eight from water, three of them from food samples and the rest were from hospital wastes.

In order to maintain aseptic condition at source, samples were collected in sterile beakers. After that, the beakers were labeled properly and transported to the laboratory as soon as possible. Furthermore, an ice box is also used for distant samples. However, as precautionary practices *e.g.*, hand gloves, masks were used during sample collection.

As environmental sources, we include soil and water sources while frozen meat and chicken nuggets were taken as food source. Moreover, pus sample, wound swab and bandage containing blood and pus were taken as clinical waste.

### 2.2 Enrichment of Culture and Isolation of *P. aeruginosa* from Various Samples

Here, 10 gm of each frozen food sample was used which was homogenized with 10 ml sterile distilled water and then added into each 50 ml of Tryptone Soy Broth. In case of other samples, soil (10gm) and water sample (10ml) was added directly into 50 ml Tryptone Soy Broth as well as bandage sample containing blood, pus and exudates was first washed with sterile distilled water and then 10ml of that water was added to 50 ml of Tryptone Soy Broth. Each flask containing Tryptone Soy Broth was kept in a shaker incubator for overnight incubation at 150 rpm to enhance the proliferation of *P. aeruginosa.*

To isolate *P. aeruginosa*, cetrimide agar medium was used as it is a well known selective media for Pseudomonas spp. After enrichment period, 0.1 ml of enriched Tryptone Soy Broth was spread on cetrimide agar and another plate was inoculated by streaked plate technique. All the plates were incubated for 24 hours at 37°C. Isolated colonies were allowed to grow on MacConkey and nutrient agar plates of the isolates.

### 2.3 Fluorescence and Hemolytic Activity Microscopic Observation after Growth

While pure cultures on cetrimide agar plates were exposed to ultra-violet light to observe the fluorescence ability, the cultures were fluorescent. Besides this, isolated colony from cetrimide plate was inoculated on blood agar and incubated at 37^°^C for 24 hours. The following observation showed clear zones around the colonies. After that, isolated colonies were allowed to grow at 42ºC for (24-72) hours to observe the growth. A pure colony was picked from nutrient agar plate and Gram staining was performed. To microscopically ensure the shape, the arrangements of colony and gram reaction were observed under a light microscope.

### 2.4 Biochemical Identification of *Pseudomonas aeruginosa* Isolates

To elucidate the bacterium, a number of biochemical tests have been conducted . Namely, Oxidase, Catalase, TSI, Citrate utilization, MIU, MR-VP, Nitrate Reduction, Gelatin Liquefaction, Fluorescences, and Hemolytic activity were performed to identify the isolates as Pseudomonas according to the Microbiological Lab Manual [12]. The representations of the outcomes of biochemical tests are attached on supplement portion.

### 2.5 Antibiotic Susceptibility Test (AST)

To attain the AST, the standard agar-disk diffusion method known as the Kirby Bauer Method [13] was used. To determine the susceptibility of the isolated *P. aeruginosa* to antibiotics, the lists of antibiotics are used. Antibiotics with their potencies used against Pseudomonas spp. are mentioned in the following Table **1**. A suspension of Pseudomonas isolate was prepared with the help of normal saline and 0.5 McFarland was used as a standard tool to maintain the perfect turbidity of the particular organism. However, that bacterial suspension was incubated for 3 hours.

After that, sterile cotton buds were used to dip into suspension, excess fluid was removed by pushing and rotating the swab firmly against the inside of the tube above the liquid level. Following this, the swab was then used to create the lawn of bacterial suspension over the entire surface of Muller Hinton agar plates. Antibiotic disks were placed aseptically over the inoculated media surface and at the same time, spatial arrangement was maintained by means of a sterile needle within a distance of 5 mm. Then the plates were incubated for 24 hours at 37ºC. While, the incubation period was over, the plates were examined and the diameters of the clear zones were measured by a ruler in mm. The zone diameters were translated into Susceptible (S), Intermediate (I) and Resistant (R) categories according to the National Committee for Clinical Laboratory Standards (NCCLS).

## 3 RESULTS

### 3.1 Isolation of *Pseudomonas aeruginosa*

After the cultivation period of 24 hours incubation at 37ºC, typical colonies on cetrimide agar MacConkey and nutrient agar media having shown following characteristics (Table **2**) were assumed to be *P. aeruginsa* and the colony is shown in Fig. (**1**). Besides this, fluorescent activity

was found when the cultures on cetrimide agar plates were exposed to Ultra-Violet light and bright green fluorescence was observed in Fig. (**2a**). Additionally, all the samples were β-hemolytic and clear zones were observed surrounding the colonies of the isolates on blood agar plates as shown in Fig. (**2b**).

### 3.2 Antibiotic Susceptibility Pattern of *Pseudomonas* Isolates

**Fig. (3):**
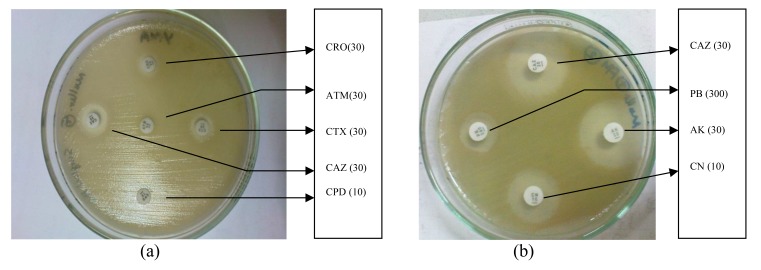
(3**a** & **b**) Antibiogram of *Pseudomonas aeruginosa*.

The Pseudomonas spp. isolated from various sources (Environmental, Food, Clinical and Hospital waste) were tested for antimicrobial susceptibility against seventeen (17) antibiotics (Fig. **3a & b**).

### 3.3 Antibiotic Susceptibility Pattern of *Pseudomonas* Isolates from Environmental Sources

Here, Fig. (**4**) indicates that 100% isolates of environmental sources were resistant to penicillin, ampicillin, and cefixime, with 90% being resistant to cefpodoxime. The above figure also depicts that 90% of the environmental isolates of *P. aeruginosa* were intermediately resistant to cefotaxime; 70%, 20% and 10% isolates were intermediately resistant to ceftriaxone, aztreonam and cefpodoxime. The figure also implies that 100% isolates showed sensitivity to levofloxacin, tobramycin, gentamicin, imipenem, piperacillin, amikacin, ciprofloxacin, meropenem and ceftazidime; 80%, 30% and 10% were sensitive to aztreonam, ceftriaxone and cefotaxime.

### 3.4 Antibiotic Susceptibility Pattern of *Pseudomonas* Isolates from Clinical Sources

From Fig. (**5**), it can be stated that 100% clinical isolates of *P.aeruginosa* were resistant to penicillin, ampicillin, cefixime, and cefpodoxime, 75% isolates were resistant to levofloxacin, tobramycin, gentamicin, and ciprofloxacin, 25% were resistant to piperacillin, amikacin, aztreonam, ceftriaxone, and cefotaxime. The above figure also implies that 75% isolates were intermediately resistant to ceftriaxone and cefotaxime, 25% were intermediately resistant to ceftazidime. The figure also clarifies that 100% isolates were sensitive to imipenem, and meropenem, 75% isolates were sensitive to piperacillin, amikacin, aztreonam, and ceftazidime, while 25% were sensitive to levofloxacin, tobramycin, gentamicin and ciprofloxacin.

### 3.5 Antibiotic Susceptibility Pattern of *Pseudomonas* Isolates from Food Sources

Fig. (**6**) indicates that 100% isolates were resistant to Penicillin, Ampicillin, Cefixime, and Cefpodoxime, while above 33% isolates were resistant to Cefotaxime. The above graph also indicates that above 66% isolates were intermediately resistant to Ceftriaxone and Cefotaxime. The above graph also implies that 100% isolates were sensitive to levofloxacin, tobramycin, gentamicin, imipenem, piperacillin, amikacin, ciprofloxacin, meropenem, aztreonam, and ceftazidime, and above 33% were sensitive to ceftriaxone.

### 3.6 Antibiotic Susceptibility Pattern of the Total Isolates of *Pseudomonas aeruginosa*

Fig. (**7**) indicates that 100% isolates were resistant to penicillin, ampicillin, cefixime, and cefpodoxime, above 17% isolates were resistant to levofloxacin, tobramycin, gentamicin and ciprofloxacin, above 11% were resistant to cefotaxime, above 5% were resistant to piperacillin, amikacin, aztreonam and ceftriaxone, The above graph also implies that 82.35%, 70.59%, 11.76% and 5.88% isolates were intermediately resistant to cefotaxime, ceftriaxone, aztreonam and ceftazidime, respectively. The figure also indicates that 100% of the total isolates were sensitive to imipenem, meropenem, above 94% were sensitive to piperacillin, amikacin and ceftazidime, above 82% of the isolates were sensitive to levofloxacin, tobramycin, gentamicin, ciprofloxacin and aztreonam, while above 23% and 5% were sensitive to ceftriaxone and cefotaxime respectively.

## 4 DISCUSSION

From the above findings it can be said that, *Pseudomonas aeruginosa* is a Gram-negative, asporogenous, obligate aerobic, motile and oxidase positive bacilli, usually found in the intestinal tract, water, soil and sewage [14] availing pathogenic potentials to unveil among individuals with the compromised immune system [15]. It is known as one of the major classes of bacterium that causes pneumonia and spreads mainly through hospital equipment’s and healthcare workers rather than interpersonal proximity [16, 17]. Frequent contamination of ventilators and hospitals’ equipments is attributed to the fact that they are resistant to temperature extremes and exposure to air . The infection could be invasive or toxicogenic [18] as well as this bacterium has a predilection for growth in moist environments, which is probably a reflection of its natural existence in soil and water [19].

This investigation implies that a total seventeen *P. aeruginosa* strains were isolated from four different sources *i.e* water sources like Tap Water (TW), Groundwater (GW), Pondwater (PW), Sewage Water (SW), river water and mineral water, soil samples from gardens, wound swab and surgical pus were the clinical samples, bandage was collected as hospital waste and three frozen food items were also included. All the isolates were tested against sixteen antibiotics to determine and compare the antibiotic susceptibility patterns. The *P. aeruginosa* strains isolated from frozen food samples were sensitive to aminoglycosides, fluoroquinolones, carboxypenicillin, carbapenems, monobactam and ceftazidime. The strains were totally resistant to penicillin, cefixime and cefpodoxime and intermediately resistant to ceftriaxone and cefotaxime. Furthermore, the environmental isolates of *P. aeruginosa* showed a similar antibiotic sensitivity pattern. Moreover, 70% and 90% of the isolates were also intermediately resistant to ceftriaxone and cefotaxime, respectively. Besides this, clinical isolates (including the isolate from medical waste material) were found to be more resistant to antibiotics than the environmental and food isolates. This might be due to the indiscriminate use of antibiotics by local physicians. The isolates were multi-drug resistant. Piperacillin, imipenem and meropenem were most the active antibiotics against the *P. aeruginosa* isolates. The second most active antibiotics were amikacin, ceftriaxone, cefotaxime and aztreonam which were 25% resistant.

A total of 5 antibiotics (aztreonam, cefpodoxime, ceftriaxone, cefotaxime and ceftazidime) were used to detect ESBL (Extended Spectrum Beta Lctamase) producing organisms [20, 21]. Among these antibiotics, all the isolates showed resistance to cefpodoxime. One of the clinical isolates showed resistance to all antibiotics used in this study except ceftazidime. It was found intermediately resistant to the antibiotic, *i.e.* ceftazidime. It could be assumed that this isolate might be an ESBL producing organism but confirmation test must be done to be certain [22].

In the present experiment, imipenem and meropenem were observed to be the most potent antibiotics (100% sensitive) followed by piperacillin, amikacin, and ceftazidime (94.12% sensitivity). 82.35% isolates were found to be sensitive to aztreonam levofloxacin, tobramycin, gentamicin and ciprofloxacin. Among 17 isolates, 70.59% of the isolates were intermediately resistant to ceftriaxone and cefotaxime. All the isolates were totally resistant to penicillin, ampicillin, and cefixime, cefpodoxime.

Here we have seen mounting evidence that the proportion of resistance (%R) among *P. aeruginosa* isolates is increasing steadily. The rise in the rate of antimicrobial resistance to a specific antibiotic was greatest for ciprofloxacin, showing an absolute increase of 16% [11]. In addition to being intrinsically resistant, it can acquire resistance trait during therapy through an array of mechanisms. The previous study in 2000-2001, in Bangladesh, showed that %R of *P. aeruginosa* to ciprofloxacin was 62.5%, ceftriaxone 75% and ceftazidime 37% [10]. However, in another study, the resistance to amikacin was 2%, for ceftriaxone, it is 43%, for ceftazidime, it is 25% and for ciprofloxacin, it is 50% [11].

The number of multi-drug resistant strains has increased in recent years. In a study conducted in the year 2003, they reported worse resistance pattern than this study, where %R to gentamycin was 93.7%, ceftazidime 96%, amikacin 93% and ciprofloxacin 86% [23]. A five-year retrospective Indian study conducted from 1997-2002 found resistance pattern of *P. aeruginosa* as amikacin to be 52%, gentamycin 69%, ciprofloxacin 89%, and ceftazidime 62%, which showed that the trend of resistance at that time was also increasing. The pattern of resistance was found to be 90% each for ceftazidime and amikacin, while 45% for ciprofloxacin [24].

The relatively high resistance of *P. aeruginosa* isolates to commonly used antibiotics as observed in this study is worrisome, especially in the developing countries like Bangladesh, where most of these antibiotics still serve as first-line drugs. From the present study, it can be revealed that environmental isolates are still sensitive to most of the commonly used antibiotics. On the other hand, since there are not proper disposal system of medical wastes in Bangladesh, there is a huge risk that the drug-resistant isolates may transmit to environmental sources using different mechanisms such as horizontal gene transfer.

## CONCLUSION

To sum up the recent study, resistance to one antibiotic is a marker for resistance to others. Antibiotic resistance is progressive, increasing from low to intermediate to high levels. However, antibiotics used by one person also affect others in the immediate and extended environment. The regretting matter is that once antibiotic resistance develops, it declines slowly since no counter selective measures exist. After all, the original, susceptible strains of organisms will only reemerge over time if they are not continuously exposed to the antibiotics(s) to which they have developed resistance.

## Figures and Tables

**Fig. (1):**
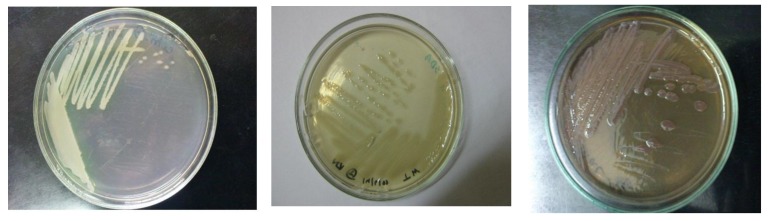
Colony Characteristics of *Pseudomonas aeruginosa* on Cetrimide agar, Nutrient agar and MacConkey agar.

**Fig. (2):**
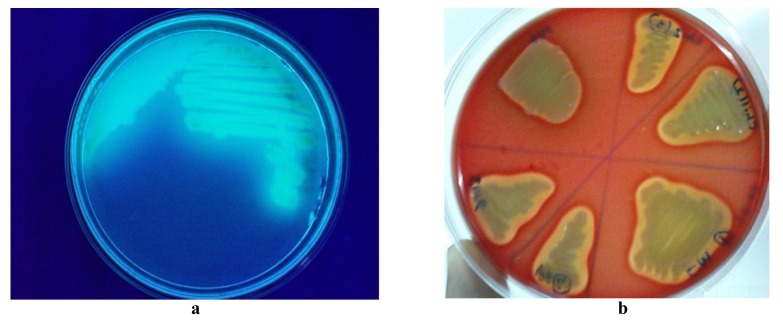
**(a)** Fluorescence activity (when exposed to UV-trans illuminator). **(b)** Hemolytic activity on blood agar shown by *P. aeruginosa*.

**Fig. (4):**
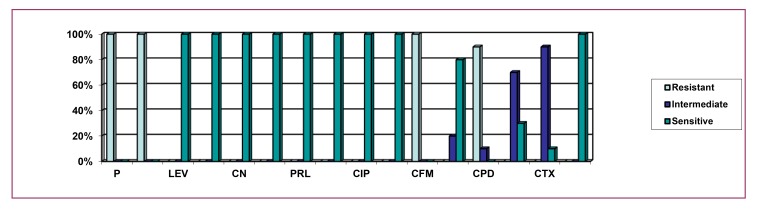
Antibiotic susceptibility pattern of *Pseudomonas aeruginosa* from Environmental sources.

**Fig. (5):**
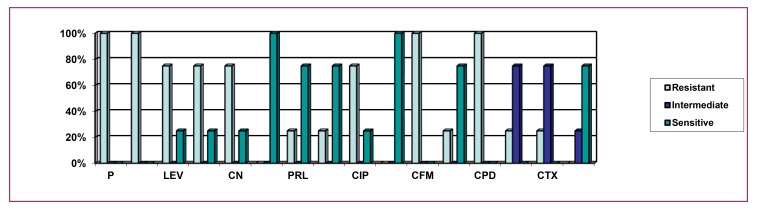
Antibiotic susceptibility pattern of *Pseudomonas aeruginosa* from Clinical samples.

**Fig. (6):**
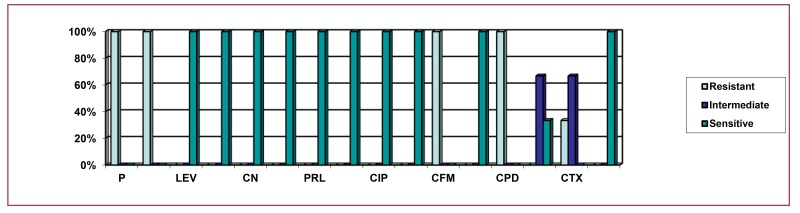
Antibiotic susceptibility pattern of *Pseudomonas aeruginosa* from Food isolates.

**Fig. (7):**
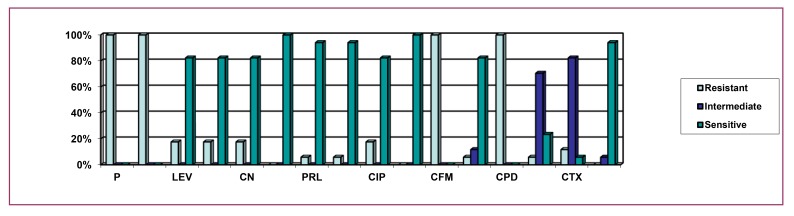
Antibiotic susceptibility pattern of total isolates of *Pseudomonas aeruginosa*.

**Table 1:** Antibiotic disks and their potencies used in the experiment.

**Antibiotics**	**Penicillin (P)**	**Ampicillin (AMP)**	**Levofloxacin (LEV)**	**Tobramycin (TOB)**	**Gentamicin (CN)**	**Imipenem (IPM)**	**Piperacillin (PRL)**	**Amikacin (AK)**
**Potencies (µg)**	10	10	5	10	10	10	75	30
**Antibiotics**	**Ciprofloxacin (CIP)**	**Meropenem (MEM)**	**Cefixime (CFM)**	**Aztreonam (ATM)**	**Cefpodoxime (CPD)**	**Ceftriaxone (CRO)**	**Cefotaxime (CTX)**	**Ceftazidime (CAZ)**
**Potencies (µg)**	5	10	5	30	10	30	30	30

**Table 2:** Colony characteristics on CetrimideAgar, Nutrient agar, MacConkey Agar media Cetrimide agar Nutrient agar MacConkey agar.

**Media**	**Colony Morphology**
Cetrimide Agar	Shiny, opaque, shiny, convex smooth,greenish-yellow colony Medium turned light blue
Nutrient Agar	Abundant, opaque, shiny, smooth, convex
MacConkey Agar	Abundant, opaque, smooth, mucoid
