# Supplementary material for: Enumerating Antibiotic Susceptibility Patterns of Pseudomonas aeruginosa Isolated from Different Sources in Dhaka City
Source: Open Microbiol J. 2018 May 31;12:172–80. doi: 10.2174/1874285801812010172 (PMC5997856; doi:10.2174/1874285801812010172)
Supplement: Supplementary file 1 [file TOMICROJ-12-172_SD.pdf]

Send Orders for Reprints to reprints@benthamscience.ae

The Open Microbiology Journal, 2018, *12*, i-iii

BENTHAM OPEN

CrossMark

The Open Microbiology Journal

Supplementary Material

Content list available at: www.benthamopen.com/TOMICROJ/

DOI: 10.2174/1874285801812010172

Enumerating Antibiotic Susceptibility Patterns of *Pseudomonas aeruginosa* Isolated from Different Sources in Dhaka City

Mahmudullah Bhuiya[1], Mohammad K. I. Sarkar[2], Mehadi H. Sohag[3], Hafij Ali[2,*], Chapol K. Roy[4], Lutfa Akther[5] and Abu F. Sarker[6]

[1]*Stamford University, Bangladesh*
[2]*Independent University, Bangladesh (IUB), Bashundhara R/A, Dhaka-1212, Bangladesh*
[3]*Jagannath University, Bangladesh*
[4]*Bangladesh Council of Scientific and Industrial Research (BCSIR), Bangladesh*
[5]*Dhaka Medical College Hospital, Bangladesh*
[6]*Rajshahi University, Bangladesh*

Received: February 15, 2018 Revised: May 04, 2018 Accepted: May 07, 2018

Microscopic Observation of *Pseudomonas aeruginosa*

All the isolates were Gram-negative, short rod shaped which are similar to *Pseudomonas* spp.

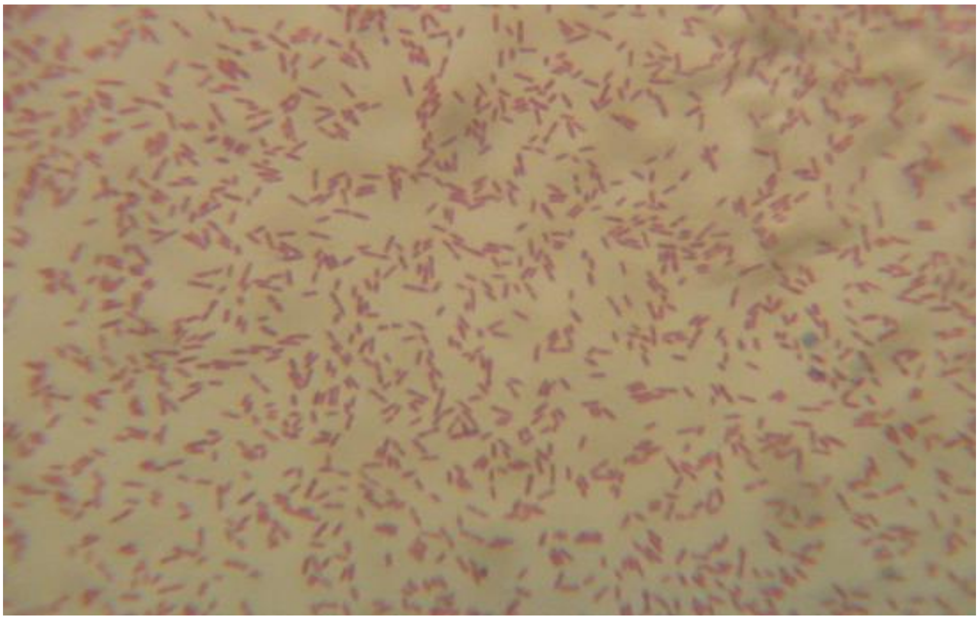

Fig. (1). Gram-staining of the of *Pseudomonas* isolate.

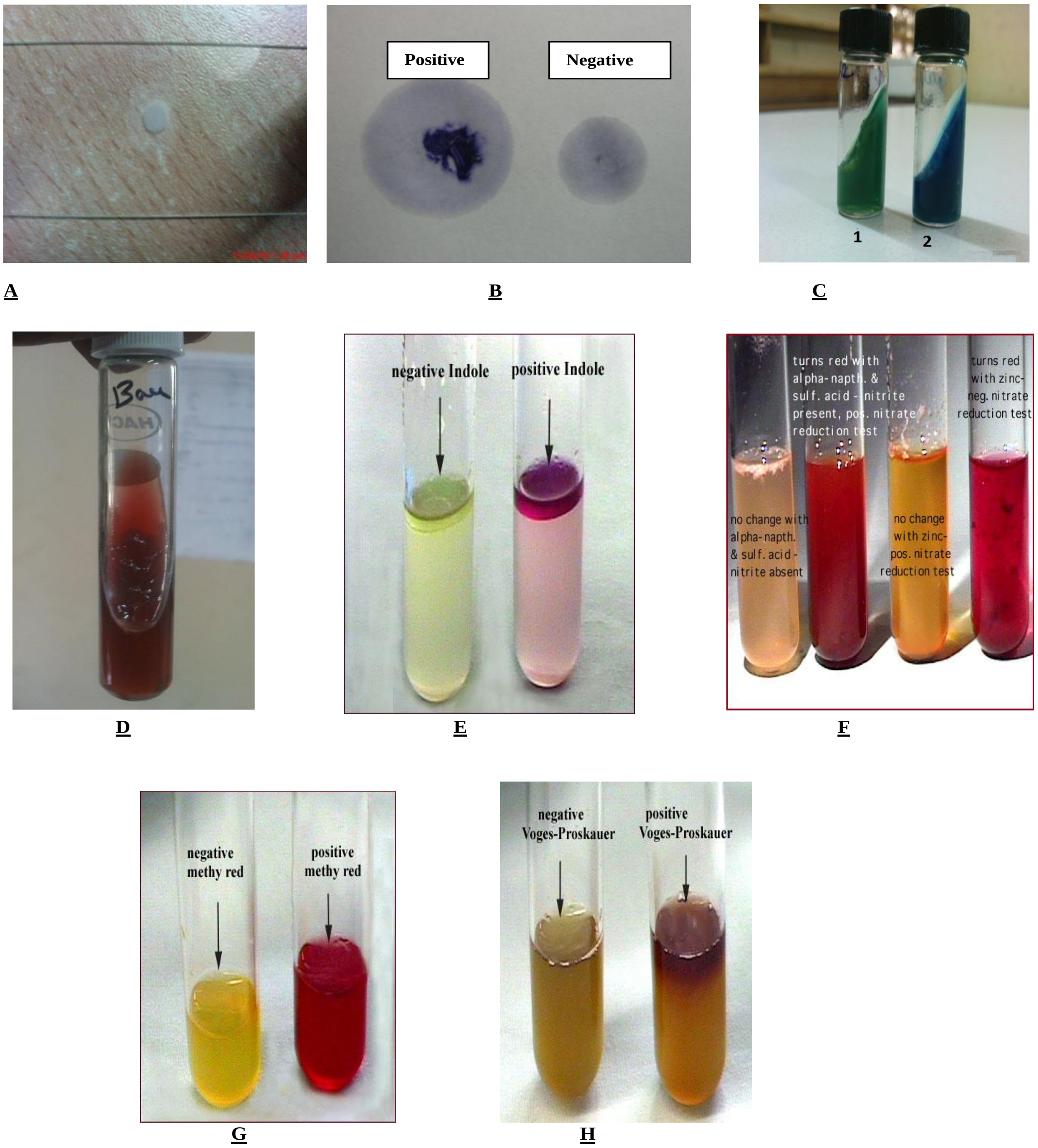

Fig. (2). Biochemical reaction test (A) Catalase test, (B) Oxidase test, (C) Citrate utilization test (1, negative; 2, positive), (D) TSI test (1, Alkaline; 2, alkaline), (E) Indole test, (F) Nitrate reduction test, (G) MR test, (H) VP test.

Biochemical Identification of *Pseudomonas aeruginosa*

Extensive biochemical tests were performed to determine the biochemical behaviour of the isolates. The results are shown in the Tables (**1**, **2** and **3**).

Table 1. Biochemical characteristics of *P. aeruginosa* (Environmental Isolates).

Strain ID	Oxidase	Catalase	Citrate Utlizatio	TSI			MIU			MR-VP		Nitrate Reduction	Gelatin Liquefaction	Fluorescence activity	Hemolytic activity	Growth at 42°C
				Slant	Butt	H_2S	M	I	U	MR	VP					
TW	+	+	+	K	K	_	+	_	+	_	_	+	+	+	β	+
PS1	+	+	+	K	K	_	+	_	+	_	_	+	+	+	β	+

(Table 1) contd.....

Strain ID	Oxidase	Catalase	Citrate Utlizatio	TSI			MIU			MR-VP		Nitrate Reduction	Gelatin Liquefaction	Fluorescence activity	Hemolytic activity	Growth at 42°C
				Slant	Butt	H_2S	M	I	U	MR	VP					
PS2	+	+	+	K	K	_	+	_	+	_	_	+	+	+	β	+
GW	+	+	+	K	K	_	+	_	+	_	_	+	+	+	β	+
SW	+	+	+	K	K	_	+	_	+	_	_	+	+	+	β	+
PO	+	+	+	K	K	_	+	_	+	_	_	+	+	+	β	+
BRW	+	+	+	K	K	_	+	_	+	_	_	+	+	+	β	+
DLW	+	+	+	K	K	_	+	_	+	_	_	+	+	+	β	+
MW1	+	+	+	K	K	_	+	_	+	_	_	+	+	+	β	+
MW2	+	+	+	K	K	_	+	_	+	_	_	+	+	+	β	+

Table 2. Biochemical characteristics of *P. aeruginosa* (Clinical Isolates).

Strain ID	Oxidase	Catalase	Citrate Utlizatio	TSI			MIU			MR-VP		Nitrate Reduction	Gelatin Liquefaction	Fluorescence activity	Hemolytic activity	Growth at 42°C
				Slant	Butt	H_2S	M	I	U	MR	VP					
WS	+	+	+	K	K	_	+	_	+	_	_	+	+	+	β	+
PUS	+	+	+	K	K	_	+	_	+	_	_	+	+	+	β	+
SUR.PUS	+	+	+	K	K	_	+	_	+	_	_	+	+	+	β	+
BAN	+	+	+	K	K	_	+	_	+	_	_	+	+	+	β	+

Table 3. Biochemical characteristics of *P.aeruginosa* (Food Isolates).

Strain ID	Oxidase	Catalase	Citrate Utlizatio	TSI			MIU			MR-VP		Nitrate Reduction	Gelatin Liquefaction	Fluorescence activity	Hemolytic activity	Growth at 42°C
				Slant	Butt	H_2S	M	I	U	MR	VP					
CN1	+	+	+	K	K	_	+	_	+	_	_	+	+		β	
CN2	+	+	+	K	K	_	+	_	+	_	_	+	+		β	
FM	+	+	+	K	K	_	+	_	+	_	_	+	+		β	

Symbols: K: Alkaline reaction, '+': Positive, '_':Negative, β: Beta

© 2018 Bhuiya *et al*.

This is an open access article distributed under the terms of the Creative Commons Attribution 4.0 International Public License (CC-BY 4.0), a copy of which is available at: (https://creativecommons.org/licenses/by/4.0/legalcode). This license permits unrestricted use, distribution, and reproduction in any medium, provided the original author and source are credited.
